# Asthma diagnosis and treatment – 1017. Prevalence and determinants of allergy and asthma amongst Indian children

**DOI:** 10.1186/1939-4551-6-S1-P17

**Published:** 2013-04-23

**Authors:** Sheetu Singh, Virendra Singh

**Affiliations:** 1 Division of Allergy & Pulmonary Medicine, SMS Medical College & Hospital, Jaipur, India

## Background

Jaipur was one of the centres that participated in the third phase of International study of asthma and allergy in children (ISAAC). The findings of Jaipur centre are highlighted in this paper. Prevalence of symptoms of asthma was assessed in school going children of Jaipur. The effects of traffic pollution and tobacco smoke on the prevalence of asthma were also studied.

## Methods

Schools at various centres were randomly selected. Children of eligible age group were included in the study after confirming with the school records. Two groups of children aged between 6-7 year and 13-14 year were selected and questionnaires were given. Parents of the younger age group children answered the questionnaire. The 13-14 year aged children answered the questions themselves along with an additional video questionnaire. The questionnaire included questions regarding exposure to traffic pollution and environmental tobacco smoke. Bivariate analysis was used to calculate the odds ratio.

## Results

The prevalence of wheeze in past 12 months (current wheeze) in the 6-7 yr age group was 5.43% and in the 13-14 yr age group was 5.37%. The prevalence of severe asthma was 3.42% in the 6-7y age group and 2.89% in the 13-14 yr age group. The proportion of asthmatics with severe asthma was high with 62.94% in the 6-7y age group and 53.85% in the older aged children. In the 6-7y age group asthma was significantly associated with environmental tobacco smoke and in the 13-14y age group asthma was associated with traffic pollution. Odds of developing bronchial asthma in the 13-14y group exposed to traffic pollution was 1.730 (95% CI: 1.209 to 2.475 ). Current wheeze was also associated in the 6-7 yr age group with maternal cigarette smoking with odd ratio of 2.627 (95% CI: 1.781 to 3.875 ) and paternal cigarette smoking with odd ratio of 9.144 (95% CI: 5.457 to 15.324).

## Conclusions

Environmental tobacco smoke and traffic pollution were associated with high prevalence of asthma amongst school going children of Jaipur.

